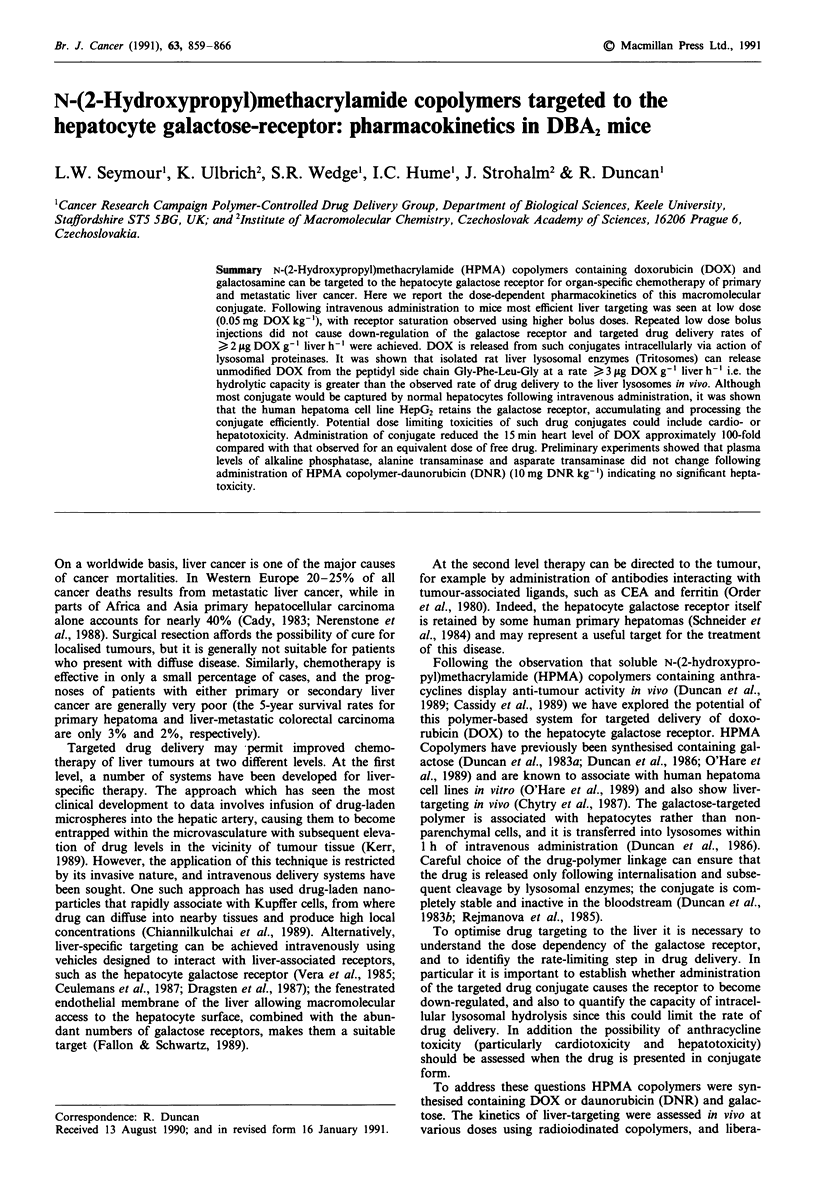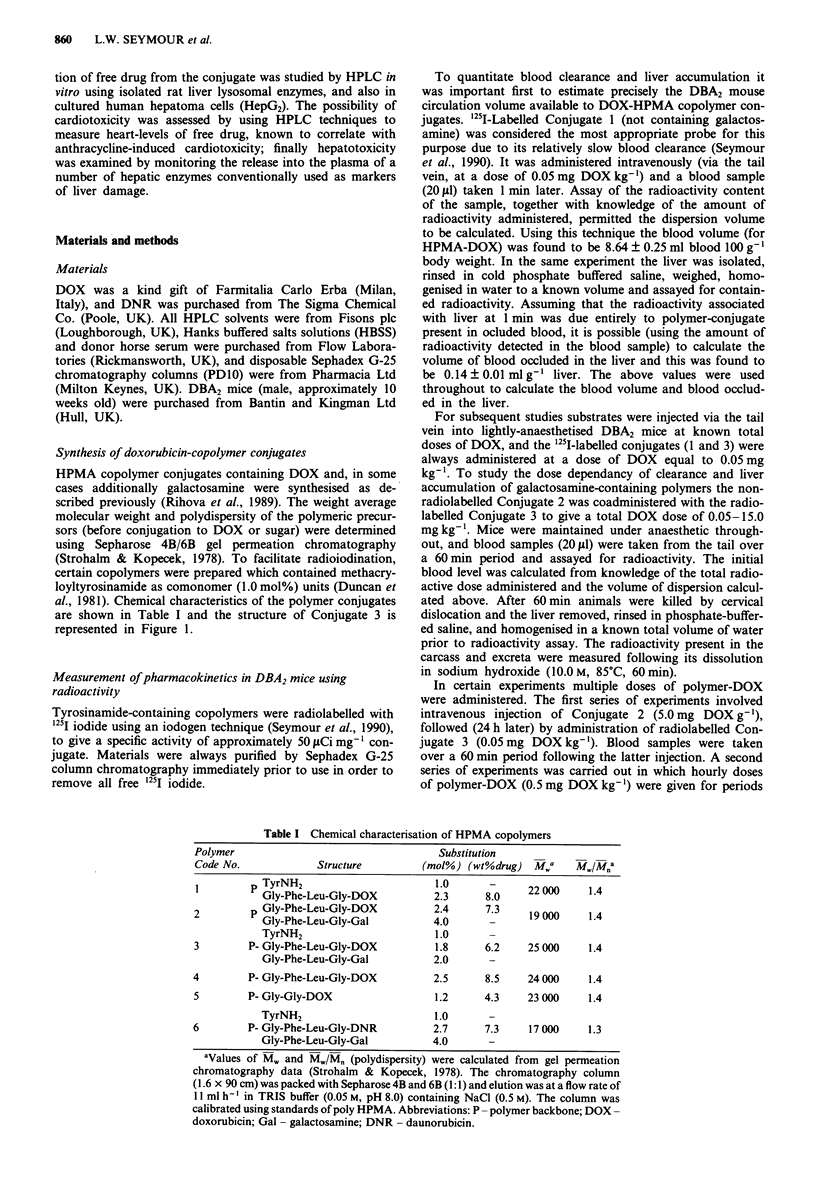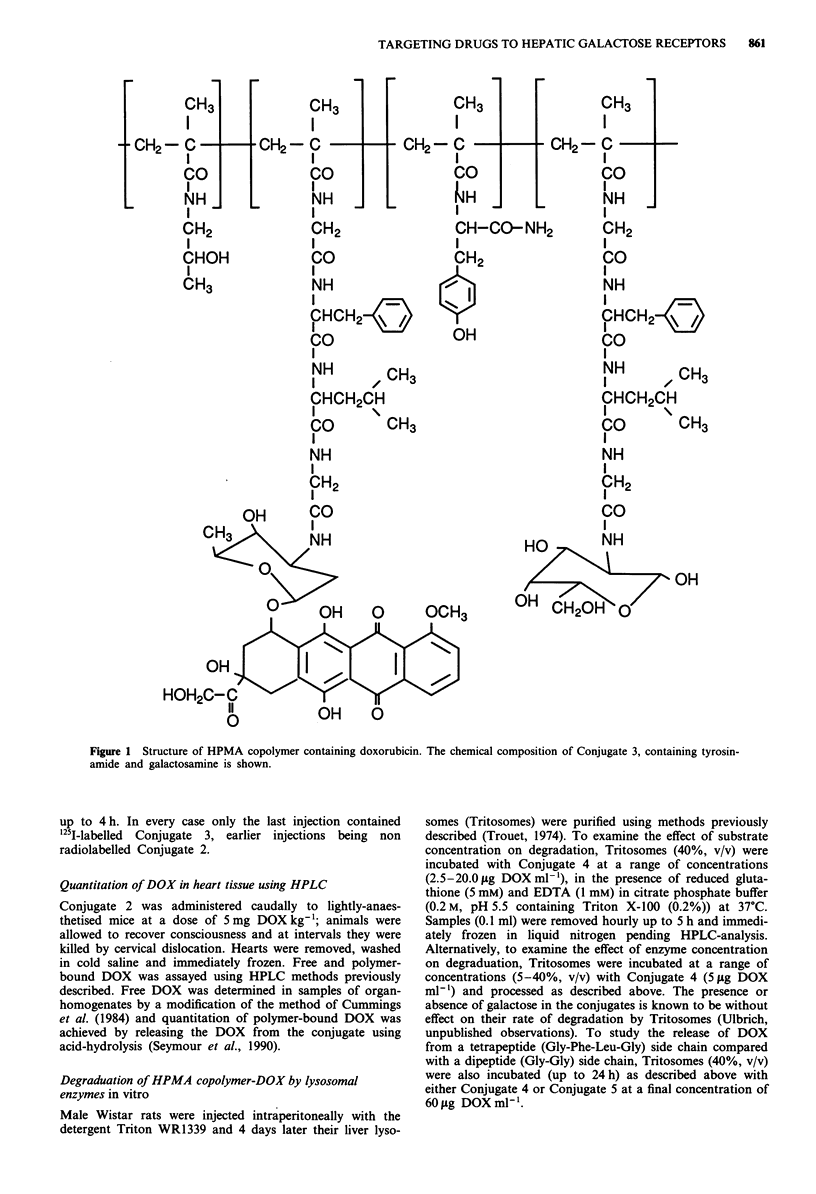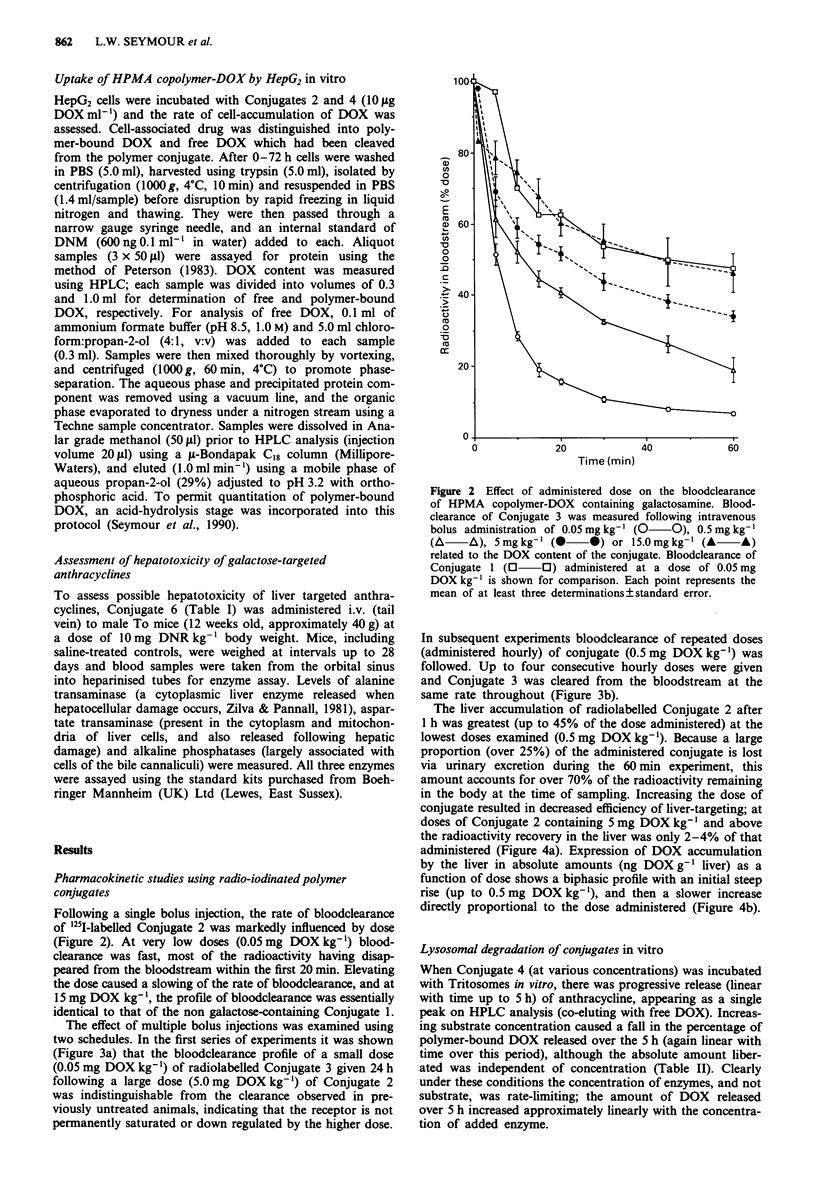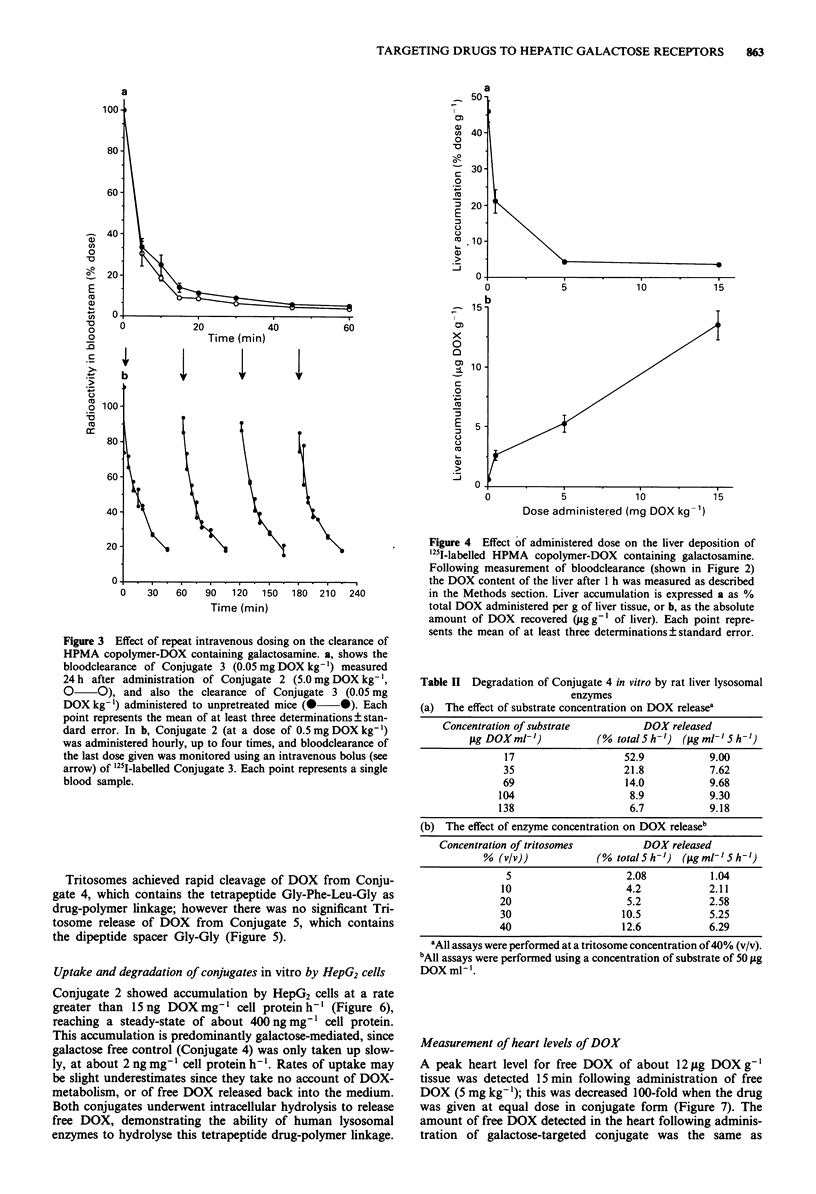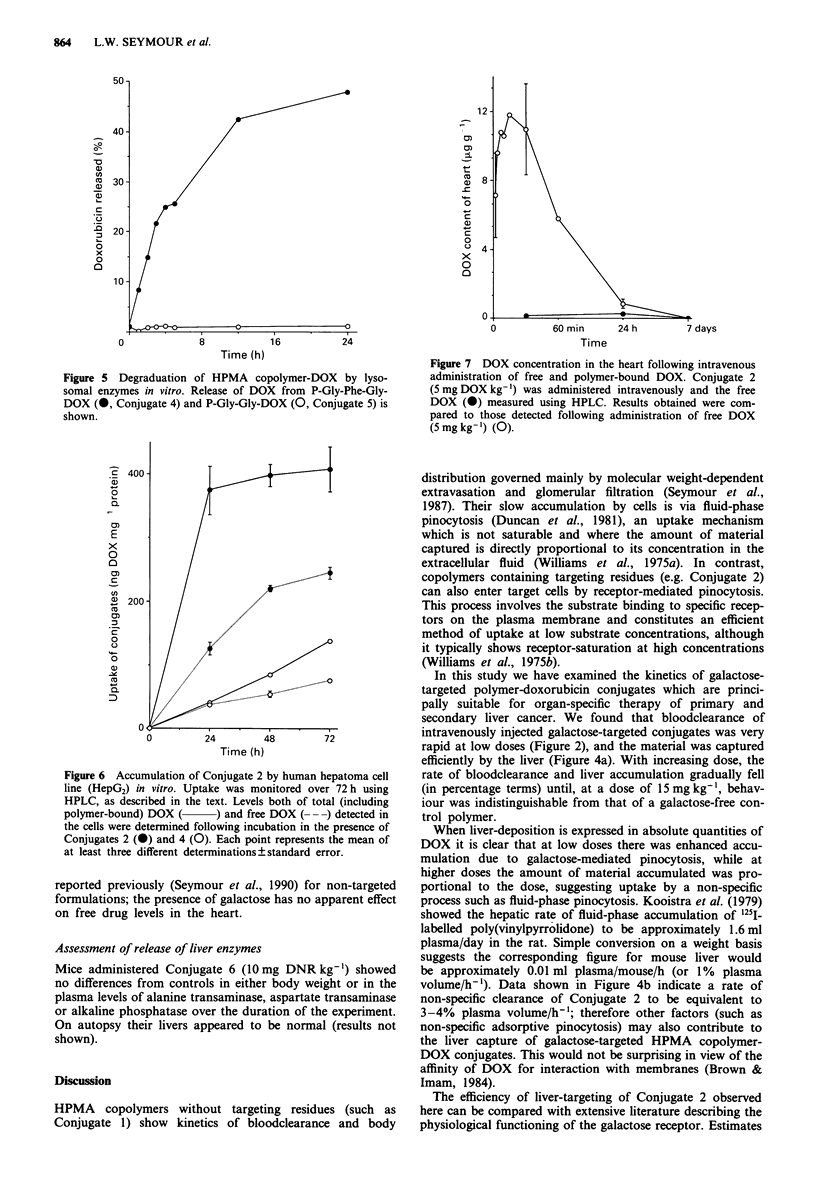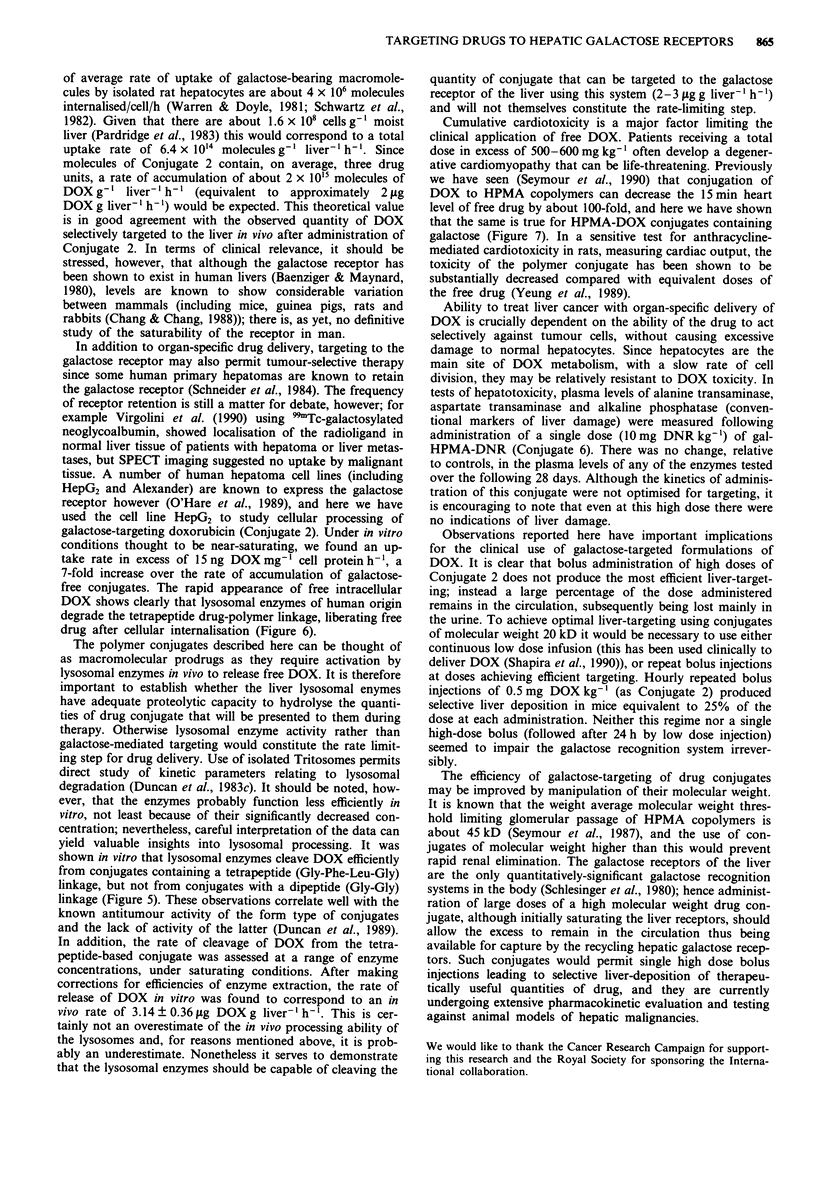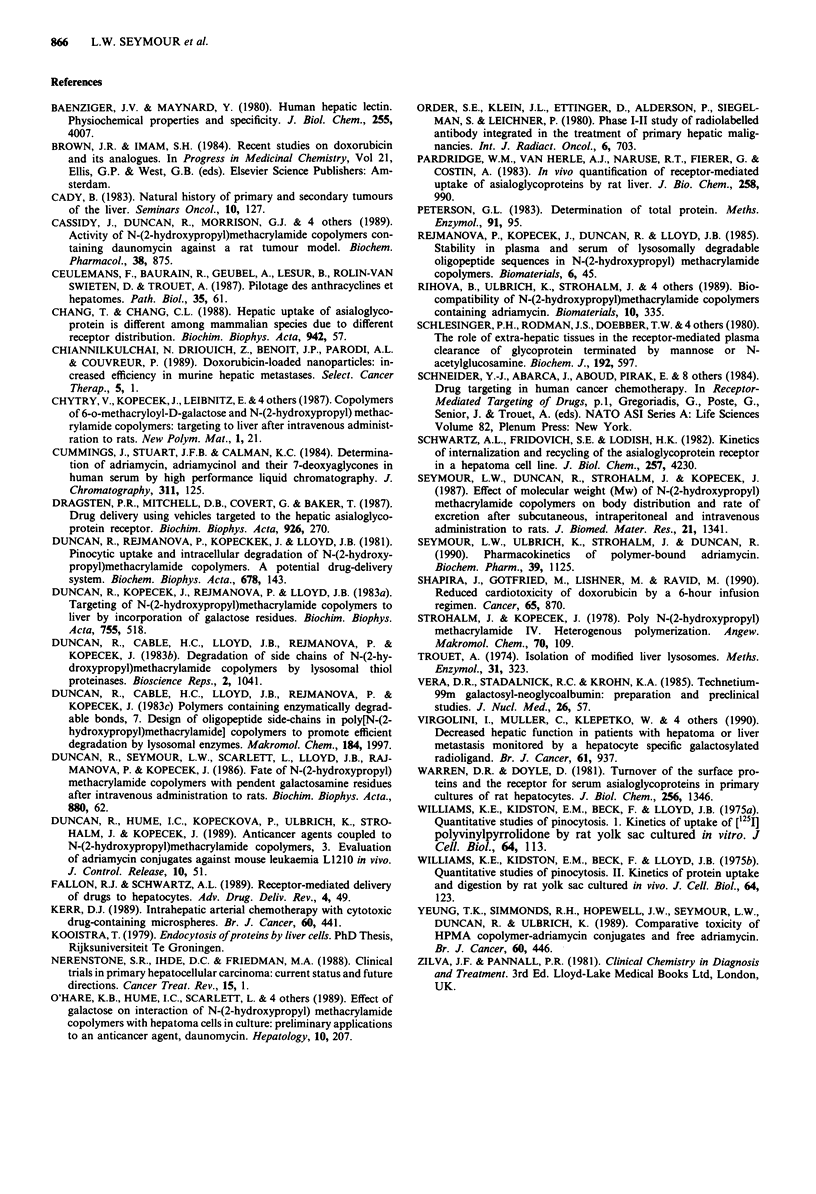# N-(2-hydroxypropyl)methacrylamide copolymers targeted to the hepatocyte galactose-receptor: pharmacokinetics in DBA2 mice.

**DOI:** 10.1038/bjc.1991.190

**Published:** 1991-06

**Authors:** L. W. Seymour, K. Ulbrich, S. R. Wedge, I. C. Hume, J. Strohalm, R. Duncan

**Affiliations:** Department of Biological Sciences, Keele University, Staffordshire, UK.

## Abstract

N-(2-Hydroxypropyl)methacrylamide (HPMA) copolymers containing doxorubicin (DOX) and galactosamine can be targeted to the hepatocyte galactose receptor for organ-specific chemotherapy of primary and metastatic liver cancer. Here we report the dose-dependent pharmacokinetics of this macromolecular conjugate. Following intravenous administration to mice most efficient liver targeting was seen at low dose (0.05 mg DOX kg-1), with receptor saturation observed using higher bolus doses. Repeated low dose bolus injections did not cause down-regulation of the galactose receptor and targeted drug delivery rates of greater than or equal to 2 micrograms DOX g-1 liver h-1 were achieved. DOX is released from such conjugates intracellularly via action of lysosomal proteinases. It was shown that isolated rat liver lysosomal enzymes (Tritosomes) can release unmodified DOX from the peptidyl side chain Gly-Phe-Leu-Gly at a rate greater than or equal to 3 micrograms DOX g-1 liver h-1 i.e. the hydrolytic capacity is greater than the observed rate of drug delivery to the liver lysosomes in vivo. Although most conjugate would be captured by normal hepatocytes following intravenous administration, it was shown that the human hepatoma cell line HepG2 retains the galactose receptor, accumulating and processing the conjugate efficiently. Potential dose limiting toxicities of such drug conjugates could include cardio- or hepatotoxicity. Administration of conjugate reduced the 15 min heart level of DOX approximately 100-fold compared with that observed for an equivalent dose of free drug. Preliminary experiments showed that plasma levels of alkaline phosphatase, alanine transaminase and asparate transaminase did not change following administration of HPMA copolymer-daunorubicin (DNR) (10 mg DNR kg-1) indicating no significant heptatoxicity.


*Br. J. Cancer* (1991), **63**, 859–866 © Macmillan Press Ltd., 1991

### N-(2-Hydroxypropyl)methacrylamide copolymers targeted to the hepatocyte galactose-receptor: pharmacokinetics in DBA$_2$ mice

L.W. Seymour[1], K. Ulbrich[2], S.R. Wedge[1], I.C. Hume[1], J. Strohalm[2] & R. Duncan[1]

[1]*Cancer Research Campaign Polymer-Controlled Drug Delivery Group, Department of Biological Sciences, Keele University, Staffordshire ST5 5BG, UK; and* [2]*Institute of Macromolecular Chemistry, Czechoslovak Academy of Sciences, 16206 Prague 6, Czechoslovakia.*

**Summary** N-(2-Hydroxypropyl)methacrylamide (HPMA) copolymers containing doxorubicin (DOX) and galactosamine can be targeted to the hepatocyte galactose receptor for organ-specific chemotherapy of primary and metastatic liver cancer. Here we report the dose-dependent pharmacokinetics of this macromolecular conjugate. Following intravenous administration to mice most efficient liver targeting was seen at low dose (0.05 mg DOX kg$^{-1}$), with receptor saturation observed using higher bolus doses. Repeated low dose bolus injections did not cause down-regulation of the galactose receptor and targeted drug delivery rates of $\geqslant 2\,\mu g$ DOX g$^{-1}$ liver h$^{-1}$ were achieved. DOX is released from such conjugates intracellularly via action of lysosomal proteinases. It was shown that isolated rat liver lysosomal enzymes (Tritosomes) can release unmodified DOX from the peptidyl side chain Gly-Phe-Leu-Gly at a rate $\geqslant 3\,\mu g$ DOX g$^{-1}$ liver h$^{-1}$ i.e. the hydrolytic capacity is greater than the observed rate of drug delivery to the liver lysosomes *in vivo*. Although most conjugate would be captured by normal hepatocytes following intravenous administration, it was shown that the human hepatoma cell line HepG$_2$ retains the galactose receptor, accumulating and processing the conjugate efficiently. Potential dose limiting toxicities of such drug conjugates could include cardio- or hepatotoxicity. Administration of conjugate reduced the 15 min heart level of DOX approximately 100-fold compared with that observed for an equivalent dose of free drug. Preliminary experiments showed that plasma levels of alkaline phosphatase, alanine transaminase and asparate transaminase did not change following administration of HPMA copolymer-daunorubicin (DNR) (10 mg DNR kg$^{-1}$) indicating no significant heptatoxicity.

On a worldwide basis, liver cancer is one of the major causes of cancer mortalities. In Western Europe 20–25% of all cancer deaths results from metastatic liver cancer, while in parts of Africa and Asia primary hepatocellular carcinoma alone accounts for nearly 40% (Cady, 1983; Nerenstone *et al.*, 1988). Surgical resection affords the possibility of cure for localised tumours, but it is generally not suitable for patients who present with diffuse disease. Similarly, chemotherapy is effective in only a small percentage of cases, and the prognoses of patients with either primary or secondary liver cancer are generally very poor (the 5-year survival rates for primary hepatoma and liver-metastatic colorectal carcinoma are only 3% and 2%, respectively).

Targeted drug delivery may permit improved chemotherapy of liver tumours at two different levels. At the first level, a number of systems have been developed for liver-specific therapy. The approach which has seen the most clinical development to data involves infusion of drug-laden microspheres into the hepatic artery, causing them to become entrapped within the microvasculature with subsequent elevation of drug levels in the vicinity of tumour tissue (Kerr, 1989). However, the application of this technique is restricted by its invasive nature, and intravenous delivery systems have been sought. One such approach has used drug-laden nanoparticles that rapidly associate with Kupffer cells, from where drug can diffuse into nearby tissues and produce high local concentrations (Chiannilkulchai *et al.*, 1989). Alternatively, liver-specific targeting can be achieved intravenously using vehicles designed to interact with liver-associated receptors, such as the hepatocyte galactose receptor (Vera *et al.*, 1985; Ceulemans *et al.*, 1987; Dragsten *et al.*, 1987); the fenestrated endothelial membrane of the liver allowing macromolecular access to the hepatocyte surface, combined with the abundant numbers of galactose receptors, makes them a suitable target (Fallon & Schwartz, 1989).

Correspondence: R. Duncan
Received 13 August 1990; and in revised form 16 January 1991.

At the second level therapy can be directed to the tumour, for example by administration of antibodies interacting with tumour-associated ligands, such as CEA and ferritin (Order *et al.*, 1980). Indeed, the hepatocyte galactose receptor itself is retained by some human primary hepatomas (Schneider *et al.*, 1984) and may represent a useful target for the treatment of this disease.

Following the observation that soluble N-(2-hydroxypropyl)methacrylamide (HPMA) copolymers containing anthracyclines display anti-tumour activity *in vivo* (Duncan *et al.*, 1989; Cassidy *et al.*, 1989) we have explored the potential of this polymer-based system for targeted delivery of doxorubicin (DOX) to the hepatocyte galactose receptor. HPMA Copolymers have previously been synthesised containing galactose (Duncan *et al.*, 1983*a*; Duncan *et al.*, 1986; O'Hare *et al.*, 1989) and are known to associate with human hepatoma cell lines *in vitro* (O'Hare *et al.*, 1989) and also show liver-targeting *in vivo* (Chytry *et al.*, 1987). The galactose-targeted polymer is associated with hepatocytes rather than non-parenchymal cells, and it is transferred into lysosomes within 1 h of intravenous administration (Duncan *et al.*, 1986). Careful choice of the drug-polymer linkage can ensure that the drug is released only following internalisation and subsequent cleavage by lysosomal enzymes; the conjugate is completely stable and inactive in the bloodstream (Duncan *et al.*, 1983*b*; Rejmanova *et al.*, 1985).

To optimise drug targeting to the liver it is necessary to understand the dose dependency of the galactose receptor, and to identifiy the rate-limiting step in drug delivery. In particular it is important to establish whether administration of the targeted drug conjugate causes the receptor to become down-regulated, and also to quantify the capacity of intracellular lysosomal hydrolysis since this could limit the rate of drug delivery. In addition the possibility of anthracycline toxicity (particularly cardiotoxicity and hepatotoxicity) should be assessed when the drug is presented in conjugate form.

To address these questions HPMA copolymers were synthesised containing DOX or daunorubicin (DNR) and galactose. The kinetics of liver-targeting were assessed *in vivo* at various doses using radioiodinated copolymers, and liberation of free drug from the conjugate was studied by HPLC *in vitro* using isolated rat liver lysosomal enzymes, and also in cultured human hepatoma cells ($HepG_2$). The possibility of cardiotoxicity was assessed by using HPLC techniques to measure heart-levels of free drug, known to correlate with anthracycline-induced cardiotoxicity; finally hepatotoxicity was examined by monitoring the release into the plasma of a number of hepatic enzymes conventionally used as markers of liver damage.

### Materials and methods

### *Materials*

DOX was a kind gift of Farmitalia Carlo Erba (Milan, Italy), and DNR was purchased from The Sigma Chemical Co. (Poole, UK). All HPLC solvents were from Fisons plc (Loughborough, UK), Hanks buffered salts solutions (HBSS) and donor horse serum were purchased from Flow Laboratories (Rickmansworth, UK), and disposable Sephadex G-25 chromatography columns (PD10) were from Pharmacia Ltd (Milton Keynes, UK). $DBA_2$ mice (male, approximately 10 weeks old) were purchased from Bantin and Kingman Ltd (Hull, UK).

### *Synthesis of doxorubicin-copolymer conjugates*

HPMA copolymer conjugates containing DOX and, in some cases additionally galactosamine were synthesised as described previously (Rihova *et al.*, 1989). The weight average molecular weight and polydispersity of the polymeric precursors (before conjugation to DOX or sugar) were determined using Sepharose 4B/6B gel permeation chromatography (Strohalm & Kopecek, 1978). To facilitate radioiodination, certain copolymers were prepared which contained methacryloyltyrosinamide as comonomer (1.0 mol%) units (Duncan *et al.*, 1981). Chemical characteristics of the polymer conjugates are shown in Table I and the structure of Conjugate 3 is represented in Figure 1.

### *Measurement of pharmacokinetics in $DBA_2$ mice using radioactivity*

Tyrosinamide-containing copolymers were radiolabelled with $^{125}I$ iodide using an iodogen technique (Seymour *et al.*, 1990), to give a specific activity of approximately 50 $\mu Ci\ mg^{-1}$ conjugate. Materials were always purified by Sephadex G-25 column chromatography immediately prior to use in order to remove all free $^{125}I$ iodide.

To quantitate blood clearance and liver accumulation it was important first to estimate precisely the $DBA_2$ mouse circulation volume available to DOX-HPMA copolymer conjugates. $^{125}I$-Labelled Conjugate 1 (not containing galactosamine) was considered the most appropriate probe for this purpose due to its relatively slow blood clearance (Seymour *et al.*, 1990). It was administered intravenously (via the tail vein, at a dose of 0.05 mg DOX $kg^{-1}$) and a blood sample (20 µl) taken 1 min later. Assay of the radioactivity content of the sample, together with knowledge of the amount of radioactivity administered, permitted the dispersion volume to be calculated. Using this technique the blood volume (for HPMA-DOX) was found to be $8.64 \pm 0.25$ ml blood 100 $g^{-1}$ body weight. In the same experiment the liver was isolated, rinsed in cold phosphate buffered saline, weighed, homogenised in water to a known volume and assayed for contained radioactivity. Assuming that the radioactivity associated with liver at 1 min was due entirely to polymer-conjugate present in ocluded blood, it is possible (using the amount of radioactivity detected in the blood sample) to calculate the volume of blood occluded in the liver and this was found to be $0.14 \pm 0.01$ ml $g^{-1}$ liver. The above values were used throughout to calculate the blood volume and blood occluded in the liver.

For subsequent studies substrates were injected via the tail vein into lightly-anaesthetised $DBA_2$ mice at known total doses of DOX, and the $^{125}I$-labelled conjugates (1 and 3) were always administered at a dose of DOX equal to 0.05 mg $kg^{-1}$. To study the dose dependancy of clearance and liver accumulation of galactosamine-containing polymers the non-radiolabelled Conjugate 2 was coadministered with the radiolabelled Conjugate 3 to give a total DOX dose of 0.05–15.0 mg $kg^{-1}$. Mice were maintained under anaesthetic throughout, and blood samples (20 µl) were taken from the tail over a 60 min period and assayed for radioactivity. The initial blood level was calculated from knowledge of the total radioactive dose administered and the volume of dispersion calculated above. After 60 min animals were killed by cervical dislocation and the liver removed, rinsed in phosphate-buffered saline, and homogenised in a known total volume of water prior to radioactivity assay. The radioactivity present in the carcass and excreta were measured following its dissolution in sodium hydroxide (10.0 M, 85°C, 60 min).

In certain experiments multiple doses of polymer-DOX were administered. The first series of experiments involved intravenous injection of Conjugate 2 (5.0 mg DOX $g^{-1}$), followed (24 h later) by administration of radiolabelled Conjugate 3 (0.05 mg DOX $kg^{-1}$). Blood samples were taken over a 60 min period following the latter injection. A second series of experiments was carried out in which hourly doses of polymer-DOX (0.5 mg DOX $kg^{-1}$) were given for periods

**Table I** Chemical characterisation of HPMA copolymers

| *Polymer Code No.* | *Structure* | *Substitution (mol%)* | *(wt%drug)* | $\overline{M}_w{}^a$ | $\overline{M}_w/\overline{M}_n{}^a$ |
|---|---|---|---|---|---|
| 1 | P TyrNH$_2$ | 1.0 | – | 22 000 | 1.4 |
| | Gly-Phe-Leu-Gly-DOX | 2.3 | 8.0 | | |
| 2 | P Gly-Phe-Leu-Gly-DOX | 2.4 | 7.3 | 19 000 | 1.4 |
| | Gly-Phe-Leu-Gly-Gal | 4.0 | – | | |
| 3 | TyrNH$_2$ | 1.0 | – | | |
| | P- Gly-Phe-Leu-Gly-DOX | 1.8 | 6.2 | 25 000 | 1.4 |
| | Gly-Phe-Leu-Gly-Gal | 2.0 | – | | |
| 4 | P- Gly-Phe-Leu-Gly-DOX | 2.5 | 8.5 | 24 000 | 1.4 |
| 5 | P- Gly-Gly-DOX | 1.2 | 4.3 | 23 000 | 1.4 |
| 6 | TyrNH$_2$ | 1.0 | – | | |
| | P- Gly-Phe-Leu-Gly-DNR | 2.7 | 7.3 | 17 000 | 1.3 |
| | Gly-Phe-Leu-Gly-Gal | 4.0 | – | | |

[a]Values of $\overline{M}_w$ and $\overline{M}_w/\overline{M}_n$ (polydispersity) were calculated from gel permeation chromatography data (Strohalm & Kopecek, 1978). The chromatography column (1.6 × 90 cm) was packed with Sepharose 4B and 6B (1:1) and elution was at a flow rate of 11 ml $h^{-1}$ in TRIS buffer (0.05 M, pH 8.0) containing NaCl (0.5 M). The column was calibrated using standards of poly HPMA. Abbreviations: P – polymer backbone; DOX – doxorubicin; Gal – galactosamine; DNR – daunorubicin.

**Figure 1** Structure of HPMA copolymer containing doxorubicin. The chemical composition of Conjugate 3, containing tyrosinamide and galactosamine is shown.

up to 4 h. In every case only the last injection contained $^{125}$I-labelled Conjugate 3, earlier injections being non radiolabelled Conjugate 2.

*Quantitation of DOX in heart tissue using HPLC*

Conjugate 2 was administered caudally to lightly-anaesthetised mice at a dose of 5 mg DOX kg$^{-1}$; animals were allowed to recover consciousness and at intervals they were killed by cervical dislocation. Hearts were removed, washed in cold saline and immediately frozen. Free and polymer-bound DOX was assayed using HPLC methods previously described. Free DOX was determined in samples of organ-homogenates by a modification of the method of Cummings *et al.* (1984) and quantitation of polymer-bound DOX was achieved by releasing the DOX from the conjugate using acid-hydrolysis (Seymour *et al.*, 1990).

*Degraduation of HPMA copolymer-DOX by lysosomal enzymes* in vitro

Male Wistar rats were injected intraperitoneally with the detergent Triton WR1339 and 4 days later their liver lysosomes (Tritosomes) were purified using methods previously described (Trouet, 1974). To examine the effect of substrate concentration on degradation, Tritosomes (40%, v/v) were incubated with Conjugate 4 at a range of concentrations (2.5–20.0 μg DOX ml$^{-1}$), in the presence of reduced glutathione (5 mM) and EDTA (1 mM) in citrate phosphate buffer (0.2 M, pH 5.5 containing Triton X-100 (0.2%)) at 37°C. Samples (0.1 ml) were removed hourly up to 5 h and immediately frozen in liquid nitrogen pending HPLC-analysis. Alternatively, to examine the effect of enzyme concentration on degraduation, Tritosomes were incubated at a range of concentrations (5–40%, v/v) with Conjugate 4 (5 μg DOX ml$^{-1}$) and processed as described above. The presence or absence of galactose in the conjugates is known to be without effect on their rate of degradation by Tritosomes (Ulbrich, unpublished observations). To study the release of DOX from a tetrapeptide (Gly-Phe-Leu-Gly) side chain compared with a dipeptide (Gly-Gly) side chain, Tritosomes (40%, v/v) were also incubated (up to 24 h) as described above with either Conjugate 4 or Conjugate 5 at a final concentration of 60 μg DOX ml$^{-1}$.

*Uptake of HPMA copolymer-DOX by $HepG_2$* in vitro

$HepG_2$ cells were incubated with Conjugates 2 and 4 (10 μg DOX $ml^{-1}$) and the rate of cell-accumulation of DOX was assessed. Cell-associated drug was distinguished into polymer-bound DOX and free DOX which had been cleaved from the polymer conjugate. After 0–72 h cells were washed in PBS (5.0 ml), harvested using trypsin (5.0 ml), isolated by centrifugation (1000 *g*, 4°C, 10 min) and resuspended in PBS (1.4 ml/sample) before disruption by rapid freezing in liquid nitrogen and thawing. They were then passed through a narrow gauge syringe needle, and an internal standard of DNM (600 ng 0.1 $ml^{-1}$ in water) added to each. Aliquot samples (3 × 50 μl) were assayed for protein using the method of Peterson (1983). DOX content was measured using HPLC; each sample was divided into volumes of 0.3 and 1.0 ml for determination of free and polymer-bound DOX, respectively. For analysis of free DOX, 0.1 ml of ammonium formate buffer (pH 8.5, 1.0 M) and 5.0 ml chloroform:propan-2-ol (4:1, v:v) was added to each sample (0.3 ml). Samples were then mixed thoroughly by vortexing, and centrifuged (1000 *g*, 60 min, 4°C) to promote phase-separation. The aqueous phase and precipitated protein component was removed using a vacuum line, and the organic phase evaporated to dryness under a nitrogen stream using a Techne sample concentrator. Samples were dissolved in Analar grade methanol (50 μl) prior to HPLC analysis (injection volume 20 μl) using a μ-Bondapak $C_{18}$ column (Millipore-Waters), and eluted (1.0 ml $min^{-1}$) using a mobile phase of aqueous propan-2-ol (29%) adjusted to pH 3.2 with orthophosphoric acid. To permit quantitation of polymer-bound DOX, an acid-hydrolysis stage was incorporated into this protocol (Seymour *et al*., 1990).

*Assessment of hepatotoxicity of galactose-targeted anthracyclines*

To assess possible hepatotoxicity of liver targeted anthracyclines, Conjugate 6 (Table I) was administered i.v. (tail vein) to male To mice (12 weeks old, approximately 40 g) at a dose of 10 mg DNR $kg^{-1}$ body weight. Mice, including saline-treated controls, were weighed at intervals up to 28 days and blood samples were taken from the orbital sinus into heparinised tubes for enzyme assay. Levels of alanine transaminase (a cytoplasmic liver enzyme released when hepatocellular damage occurs, Zilva & Pannall, 1981), aspartate transaminase (present in the cytoplasm and mitochondria of liver cells, and also released following hepatic damage) and alkaline phosphatases (largely associated with cells of the bile cannaliculi) were measured. All three enzymes were assayed using the standard kits purchased from Boehringer Mannheim (UK) Ltd (Lewes, East Sussex).

### Results

*Pharmacokinetic studies using radio-iodinated polymer conjugates*

Following a single bolus injection, the rate of bloodclearance of $^{125}I$-labelled Conjugate 2 was markedly influenced by dose (Figure 2). At very low doses (0.05 mg DOX $kg^{-1}$) bloodclearance was fast, most of the radioactivity having disappeared from the bloodstream within the first 20 min. Elevating the dose caused a slowing of the rate of bloodclearance, and at 15 mg DOX $kg^{-1}$, the profile of bloodclearance was essentially identical to that of the non galactose-containing Conjugate 1.

The effect of multiple bolus injections was examined using two schedules. In the first series of experiments it was shown (Figure 3a) that the bloodclearance profile of a small dose (0.05 mg DOX $kg^{-1}$) of radiolabelled Conjugate 3 given 24 h following a large dose (5.0 mg DOX $kg^{-1}$) of Conjugate 2 was indistinguishable from the clearance observed in previously untreated animals, indicating that the receptor is not permanently saturated or down regulated by the higher dose.

**Figure 2** Effect of administered dose on the bloodclearance of HPMA copolymer-DOX containing galactosamine. Bloodclearance of Conjugate 3 was measured following intravenous bolus administration of 0.05 mg $kg^{-1}$ (○——○), 0.5 mg $kg^{-1}$ (△——△), 5 mg $kg^{-1}$ (●——●) or 15.0 mg $kg^{-1}$ (▲——▲) related to the DOX content of the conjugate. Bloodclearance of Conjugate 1 (□——□) administered at a dose of 0.05 mg DOX $kg^{-1}$ is shown for comparison. Each point represents the mean of at least three determinations ± standard error.

In subsequent experiments bloodclearance of repeated doses (administered hourly) of conjugate (0.5 mg DOX $kg^{-1}$) was followed. Up to four consecutive hourly doses were given and Conjugate 3 was cleared from the bloodstream at the same rate throughout (Figure 3b).

The liver accumulation of radiolabelled Conjugate 2 after 1 h was greatest (up to 45% of the dose administered) at the lowest doses examined (0.5 mg DOX $kg^{-1}$). Because a large proportion (over 25%) of the administered conjugate is lost via urinary excretion during the 60 min experiment, this amount accounts for over 70% of the radioactivity remaining in the body at the time of sampling. Increasing the dose of conjugate resulted in decreased efficiency of liver-targeting; at doses of Conjugate 2 containing 5 mg DOX $kg^{-1}$ and above the radioactivity recovery in the liver was only 2–4% of that administered (Figure 4a). Expression of DOX accumulation by the liver in absolute amounts (ng DOX $g^{-1}$ liver) as a function of dose shows a biphasic profile with an initial steep rise (up to 0.5 mg DOX $kg^{-1}$), and then a slower increase directly proportional to the dose administered (Figure 4b).

*Lysosomal degradation of conjugates* in vitro

When Conjugate 4 (at various concentrations) was incubated with Tritosomes *in vitro*, there was progressive release (linear with time up to 5 h) of anthracycline, appearing as a single peak on HPLC analysis (co-eluting with free DOX). Increasing substrate concentration caused a fall in the percentage of polymer-bound DOX released over the 5 h (again linear with time over this period), although the absolute amount liberated was independent of concentration (Table II). Clearly under these conditions the concentration of enzymes, and not substrate, was rate-limiting; the amount of DOX released over 5 h increased approximately linearly with the concentration of added enzyme.

**Figure 3** Effect of repeat intravenous dosing on the clearance of HPMA copolymer-DOX containing galactosamine. **a**, shows the bloodclearance of Conjugate 3 (0.05 mg DOX $kg^{-1}$) measured 24 h after administration of Conjugate 2 (5.0 mg DOX $kg^{-1}$, O——O), and also the clearance of Conjugate 3 (0.05 mg DOX $kg^{-1}$) administered to unpretreated mice (●——●). Each point represents the mean of at least three determinations ± standard error. In **b**, Conjugate 2 (at a dose of 0.5 mg DOX $kg^{-1}$) was administered hourly, up to four times, and bloodclearance of the last dose given was monitored using an intravenous bolus (see arrow) of $^{125}$I-labelled Conjugate 3. Each point represents a single blood sample.

**Figure 4** Effect of administered dose on the liver deposition of $^{125}$I-labelled HPMA copolymer-DOX containing galactosamine. Following measurement of bloodclearance (shown in Figure 2) the DOX content of the liver after 1 h was measured as described in the Methods section. Liver accumulation is expressed **a** as % total DOX administered per g of liver tissue, or **b**, as the absolute amount of DOX recovered (µg $g^{-1}$ of liver). Each point represents the mean of at least three determinations ± standard error.

**Table II** Degradation of Conjugate 4 *in vitro* by rat liver lysosomal enzymes

(a) The effect of substrate concentration on DOX release[a]

| *Concentration of substrate µg DOX $ml^{-1}$)* | *DOX released (% total 5 $h^{-1}$)* | *(µg $ml^{-1}$ 5 $h^{-1}$)* |
|---|---|---|
| 17 | 52.9 | 9.00 |
| 35 | 21.8 | 7.62 |
| 69 | 14.0 | 9.68 |
| 104 | 8.9 | 9.30 |
| 138 | 6.7 | 9.18 |

(b) The effect of enzyme concentration on DOX release[b]

| *Concentration of tritosomes % (v/v))* | *DOX released (% total 5 $h^{-1}$)* | *(µg $ml^{-1}$ 5 $h^{-1}$)* |
|---|---|---|
| 5 | 2.08 | 1.04 |
| 10 | 4.2 | 2.11 |
| 20 | 5.2 | 2.58 |
| 30 | 10.5 | 5.25 |
| 40 | 12.6 | 6.29 |

[a]All assays were performed at a tritosome concentration of 40% (v/v). [b]All assays were performed using a concentration of substrate of 50 µg DOX $ml^{-1}$.

Tritosomes achieved rapid cleavage of DOX from Conjugate 4, which contains the tetrapeptide Gly-Phe-Leu-Gly as drug-polymer linkage; however there was no significant Tritosome release of DOX from Conjugate 5, which contains the dipeptide spacer Gly-Gly (Figure 5).

### *Uptake and degradation of conjugates* in vitro *by $HepG_2$ cells*

Conjugate 2 showed accumulation by $HepG_2$ cells at a rate greater than 15 ng DOX $mg^{-1}$ cell protein $h^{-1}$ (Figure 6), reaching a steady-state of about 400 ng $mg^{-1}$ cell protein. This accumulation is predominantly galactose-mediated, since galactose free control (Conjugate 4) was only taken up slowly, at about 2 ng $mg^{-1}$ cell protein $h^{-1}$. Rates of uptake may be slight underestimates since they take no account of DOX-metabolism, or of free DOX released back into the medium. Both conjugates underwent intracellular hydrolysis to release free DOX, demonstrating the ability of human lysosomal enzymes to hydrolyse this tetrapeptide drug-polymer linkage.

### *Measurement of heart levels of DOX*

A peak heart level for free DOX of about 12 µg DOX $g^{-1}$ tissue was detected 15 min following administration of free DOX (5 mg $kg^{-1}$); this was decreased 100-fold when the drug was given at equal dose in conjugate form (Figure 7). The amount of free DOX detected in the heart following administration of galactose-targeted conjugate was the same as

**Figure 5** Degraduation of HPMA copolymer-DOX by lysosomal enzymes *in vitro*. Release of DOX from P-Gly-Phe-Gly-DOX (●, Conjugate 4) and P-Gly-Gly-DOX (○, Conjugate 5) is shown.

**Figure 6** Accumulation of Conjugate 2 by human hepatoma cell line ($HepG_2$) *in vitro*. Uptake was monitored over 72 h using HPLC, as described in the text. Levels both of total (including polymer-bound) DOX (———) and free DOX (- - -) detected in the cells were determined following incubation in the presence of Conjugates 2 (●) and 4 (○). Each point represents the mean of at least three different determinations ± standard error.

reported previously (Seymour *et al.*, 1990) for non-targeted formulations; the presence of galactose has no apparent effect on free drug levels in the heart.

*Assessment of release of liver enzymes*

Mice administered Conjugate 6 (10 mg DNR $kg^{-1}$) showed no differences from controls in either body weight or in the plasma levels of alanine transaminase, aspartate transaminase or alkaline phosphatase over the duration of the experiment. On autopsy their livers appeared to be normal (results not shown).

**Discussion**

HPMA copolymers without targeting residues (such as Conjugate 1) show kinetics of bloodclearance and body

**Figure 7** DOX concentration in the heart following intravenous administration of free and polymer-bound DOX. Conjugate 2 (5 mg DOX $kg^{-1}$) was administered intravenously and the free DOX (●) measured using HPLC. Results obtained were compared to those detected following administration of free DOX (5 mg $kg^{-1}$) (○).

distribution governed mainly by molecular weight-dependent extravasation and glomerular filtration (Seymour *et al.*, 1987). Their slow accumulation by cells is via fluid-phase pinocytosis (Duncan *et al.*, 1981), an uptake mechanism which is not saturable and where the amount of material captured is directly proportional to its concentration in the extracellular fluid (Williams *et al.*, 1975*a*). In contrast, copolymers containing targeting residues (e.g. Conjugate 2) can also enter target cells by receptor-mediated pinocytosis. This process involves the substrate binding to specific receptors on the plasma membrane and constitutes an efficient method of uptake at low substrate concentrations, although it typically shows receptor-saturation at high concentrations (Williams *et al.*, 1975*b*).

In this study we have examined the kinetics of galactose-targeted polymer-doxorubicin conjugates which are principally suitable for organ-specific therapy of primary and secondary liver cancer. We found that bloodclearance of intravenously injected galactose-targeted conjugates was very rapid at low doses (Figure 2), and the material was captured efficiently by the liver (Figure 4a). With increasing dose, the rate of bloodclearance and liver accumulation gradually fell (in percentage terms) until, at a dose of 15 mg $kg^{-1}$, behaviour was indistinguishable from that of a galactose-free control polymer.

When liver-deposition is expressed in absolute quantities of DOX it is clear that at low doses there was enhanced accumulation due to galactose-mediated pinocytosis, while at higher doses the amount of material accumulated was proportional to the dose, suggesting uptake by a non-specific process such as fluid-phase pinocytosis. Kooistra *et al.* (1979) showed the hepatic rate of fluid-phase accumulation of $^{125}$I-labelled poly(vinylpyrrolidone) to be approximately 1.6 ml plasma/day in the rat. Simple conversion on a weight basis suggests the corresponding figure for mouse liver would be approximately 0.01 ml plasma/mouse/h (or 1% plasma volume/$h^{-1}$). Data shown in Figure 4b indicate a rate of non-specific clearance of Conjugate 2 to be equivalent to 3–4% plasma volume/$h^{-1}$; therefore other factors (such as non-specific adsorptive pinocytosis) may also contribute to the liver capture of galactose-targeted HPMA copolymer-DOX conjugates. This would not be surprising in view of the affinity of DOX for interaction with membranes (Brown & Imam, 1984).

The efficiency of liver-targeting of Conjugate 2 observed here can be compared with extensive literature describing the physiological functioning of the galactose receptor. Estimates of average rate of uptake of galactose-bearing macromolecules by isolated rat hepatocytes are about $4 \times 10^6$ molecules internalised/cell/h (Warren & Doyle, 1981; Schwartz *et al.*, 1982). Given that there are about $1.6 \times 10^8$ cells $g^{-1}$ moist liver (Pardridge *et al.*, 1983) this would correspond to a total uptake rate of $6.4 \times 10^{14}$ molecules $g^{-1}$ liver$^{-1}$ $h^{-1}$. Since molecules of Conjugate 2 contain, on average, three drug units, a rate of accumulation of about $2 \times 10^{15}$ molecules of DOX $g^{-1}$ liver$^{-1}$ $h^{-1}$ (equivalent to approximately 2 $\mu$g DOX g liver$^{-1}$ $h^{-1}$) would be expected. This theoretical value is in good agreement with the observed quantity of DOX selectively targeted to the liver *in vivo* after administration of Conjugate 2. In terms of clinical relevance, it should be stressed, however, that although the galactose receptor has been shown to exist in human livers (Baenziger & Maynard, 1980), levels are known to show considerable variation between mammals (including mice, guinea pigs, rats and rabbits (Chang & Chang, 1988)); there is, as yet, no definitive study of the saturability of the receptor in man.

In addition to organ-specific drug delivery, targeting to the galactose receptor may also permit tumour-selective therapy since some human primary hepatomas are known to retain the galactose receptor (Schneider *et al.*, 1984). The frequency of receptor retention is still a matter for debate, however; for example Virgolini *et al.* (1990) using $^{99m}$Tc-galactosylated neoglycoalbumin, showed localisation of the radioligand in normal liver tissue of patients with hepatoma or liver metastases, but SPECT imaging suggested no uptake by malignant tissue. A number of human hepatoma cell lines (including $HepG_2$ and Alexander) are known to express the galactose receptor however (O'Hare *et al.*, 1989), and here we have used the cell line $HepG_2$ to study cellular processing of galactose-targeting doxorubicin (Conjugate 2). Under *in vitro* conditions thought to be near-saturating, we found an uptake rate in excess of 15 ng DOX $mg^{-1}$ cell protein $h^{-1}$, a 7-fold increase over the rate of accumulation of galactose-free conjugates. The rapid appearance of free intracellular DOX shows clearly that lysosomal enzymes of human origin degrade the tetrapeptide drug-polymer linkage, liberating free drug after cellular internalisation (Figure 6).

The polymer conjugates described here can be thought of as macromolecular prodrugs as they require activation by lysosomal enzymes *in vivo* to release free DOX. It is therefore important to establish whether the liver lysosomal enymes have adequate proteolytic capacity to hydrolyse the quantities of drug conjugate that will be presented to them during therapy. Otherwise lysosomal enzyme activity rather than galactose-mediated targeting would constitute the rate limiting step for drug delivery. Use of isolated Tritosomes permits direct study of kinetic parameters relating to lysosomal degradation (Duncan *et al.*, 1983*c*). It should be noted, however, that the enzymes probably function less efficiently *in vitro*, not least because of their significantly decreased concentration; nevertheless, careful interpretation of the data can yield valuable insights into lysosomal processing. It was shown *in vitro* that lysosomal enzymes cleave DOX efficiently from conjugates containing a tetrapeptide (Gly-Phe-Leu-Gly) linkage, but not from conjugates with a dipeptide (Gly-Gly) linkage (Figure 5). These observations correlate well with the known antitumour activity of the form type of conjugates and the lack of activity of the latter (Duncan *et al.*, 1989). In addition, the rate of cleavage of DOX from the tetrapeptide-based conjugate was assessed at a range of enzyme concentrations, under saturating conditions. After making corrections for efficiencies of enzyme extraction, the rate of release of DOX *in vitro* was found to correspond to an *in vivo* rate of $3.14 \pm 0.36$ $\mu$g DOX g liver$^{-1}$ $h^{-1}$. This is certainly not an overestimate of the *in vivo* processing ability of the lysosomes and, for reasons mentioned above, it is probably an underestimate. Nonetheless it serves to demonstrate that the lysosomal enzymes should be capable of cleaving the quantity of conjugate that can be targeted to the galactose receptor of the liver using this system (2–3 $\mu$g g liver$^{-1}$ $h^{-1}$) and will not themselves constitute the rate-limiting step.

Cumulative cardiotoxicity is a major factor limiting the clinical application of free DOX. Patients receiving a total dose in excess of 500–600 mg $kg^{-1}$ often develop a degenerative cardiomyopathy that can be life-threatening. Previously we have seen (Seymour *et al.*, 1990) that conjugation of DOX to HPMA copolymers can decrease the 15 min heart level of free drug by about 100-fold, and here we have shown that the same is true for HPMA-DOX conjugates containing galactose (Figure 7). In a sensitive test for anthracycline-mediated cardiotoxicity in rats, measuring cardiac output, the toxicity of the polymer conjugate has been shown to be substantially decreased compared with equivalent doses of the free drug (Yeung *et al.*, 1989).

Ability to treat liver cancer with organ-specific delivery of DOX is crucially dependent on the ability of the drug to act selectively against tumour cells, without causing excessive damage to normal hepatocytes. Since hepatocytes are the main site of DOX metabolism, with a slow rate of cell division, they may be relatively resistant to DOX toxicity. In tests of hepatotoxicity, plasma levels of alanine transaminase, aspartate transaminase and alkaline phosphatase (conventional markers of liver damage) were measured following administration of a single dose (10 mg DNR $kg^{-1}$) of gal-HPMA-DNR (Conjugate 6). There was no change, relative to controls, in the plasma levels of any of the enzymes tested over the following 28 days. Although the kinetics of administration of this conjugate were not optimised for targeting, it is encouraging to note that even at this high dose there were no indications of liver damage.

Observations reported here have important implications for the clinical use of galactose-targeted formulations of DOX. It is clear that bolus administration of high doses of Conjugate 2 does not produce the most efficient liver-targeting; instead a large percentage of the dose administered remains in the circulation, subsequently being lost mainly in the urine. To achieve optimal liver-targeting using conjugates of molecular weight 20 kD it would be necessary to use either continuous low dose infusion (this has been used clinically to deliver DOX (Shapira *et al.*, 1990)), or repeat bolus injections at doses achieving efficient targeting. Hourly repeated bolus injections of 0.5 mg DOX $kg^{-1}$ (as Conjugate 2) produced selective liver deposition in mice equivalent to 25% of the dose at each administration. Neither this regime nor a single high-dose bolus (followed after 24 h by low dose injection) seemed to impair the galactose recognition system irreversibly.

The efficiency of galactose-targeting of drug conjugates may be improved by manipulation of their molecular weight. It is known that the weight average molecular weight threshold limiting glomerular passage of HPMA copolymers is about 45 kD (Seymour *et al.*, 1987), and the use of conjugates of molecular weight higher than this would prevent rapid renal elimination. The galactose receptors of the liver are the only quantitatively-significant galactose recognition systems in the body (Schlesinger *et al.*, 1980); hence administration of large doses of a high molecular weight drug conjugate, although initially saturating the liver receptors, should allow the excess to remain in the circulation thus being available for capture by the recycling hepatic galactose receptors. Such conjugates would permit single high dose bolus injections leading to selective liver-deposition of therapeutically useful quantities of drug, and they are currently undergoing extensive pharmacokinetic evaluation and testing against animal models of hepatic malignancies.

We would like to thank the Cancer Research Campaign for supporting this research and the Royal Society for sponsoring the International collaboration.

### References

BAENZIGER, J.V. & MAYNARD, Y. (1980). Human hepatic lectin. Physiochemical properties and specificity. *J. Biol. Chem.*, **255**, 4007.

BROWN, J.R. & IMAM, S.H. (1984). Recent studies on doxorubicin and its analogues. In *Progress in Medicinal Chemistry*, Vol 21, Ellis, G.P. & West, G.B. (eds). Elsevier Science Publishers: Amsterdam.

CADY, B. (1983). Natural history of primary and secondary tumours of the liver. *Seminars Oncol.*, **10**, 127.

CASSIDY, J., DUNCAN, R., MORRISON, G.J. & 4 others (1989). Activity of N-(2-hydroxypropyl)methacrylamide copolymers containing daunomycin against a rat tumour model. *Biochem. Pharmacol.*, **38**, 875.

CEULEMANS, F., BAURAIN, R., GEUBEL, A., LESUR, B., ROLIN-VAN SWIETEN, D. & TROUET, A. (1987). Pilotage des anthracyclines et hepatomes. *Path. Biol.*, **35**, 61.

CHANG, T. & CHANG, C.L. (1988). Hepatic uptake of asialoglycoprotein is different among mammalian species due to different receptor distribution. *Biochim. Biophys. Acta*, **942**, 57.

CHIANNILKULCHAI, N. DRIOUICH, Z., BENOIT, J.P., PARODI, A.L. & COUVREUR, P. (1989). Doxorubicin-loaded nanoparticles: increased efficiency in murine hepatic metastases. *Select. Cancer Therap.*, **5**, 1.

CHYTRY, V., KOPECEK, J., LEIBNITZ, E. & 4 others (1987). Copolymers of 6-o-methacryloyl-D-galactose and N-(2-hydroxypropyl) methacrylamide copolymers: targeting to liver after intravenous administration to rats. *New Polym. Mat.*, **1**, 21.

CUMMINGS, J., STUART, J.F.B. & CALMAN, K.C. (1984). Determination of adriamycin, adriamycinol and their 7-deoxyaglycones in human serum by high performance liquid chromatography. *J. Chromatography*, **311**, 125.

DRAGSTEN, P.R., MITCHELL, D.B., COVERT, G. & BAKER, T. (1987). Drug delivery using vehicles targeted to the hepatic asialoglycoprotein receptor. *Biochim. Biophys. Acta*, **926**, 270.

DUNCAN, R., REJMANOVA, P., KOPECKEK, J. & LLOYD, J.B. (1981). Pinocytic uptake and intracellular degradation of N-(2-hydroxypropyl)methacrylamide copolymers. A potential drug-delivery system. *Biochem. Biophys. Acta.*, **678**, 143.

DUNCAN, R., KOPECEK, J., REJMANOVA, P. & LLOYD, J.B. (1983*a*). Targeting of N-(2-hydroxypropyl)methacrylamide copolymers to liver by incorporation of galactose residues. *Biochim. Biophys. Acta*, **755**, 518.

DUNCAN, R., CABLE, H.C., LLOYD, J.B., REJMANOVA, P. & KOPECEK, J. (1983*b*). Degradation of side chains of N-(2-hydroxypropyl)methacrylamide copolymers by lysosomal thiol proteinases. *Bioscience Reps.*, **2**, 1041.

DUNCAN, R., CABLE, H.C., LLOYD, J.B., REJMANOVA, P. & KOPECEK, J. (1983*c*) Polymers containing enzymatically degradable bonds, 7. Design of oligopeptide side-chains in poly[N-(2-hydroxypropyl)methacrylamide] copolymers to promote efficient degradation by lysosomal enzymes. *Makromol. Chem.*, **184**, 1997.

DUNCAN, R., SEYMOUR, L.W., SCARLETT, L., LLOYD, J.B., RAJMANOVA, P. & KOPECEK, J. (1986). Fate of N-(2-hydroxypropyl) methacrylamide copolymers with pendent galactosamine residues after intravenous administration to rats. *Biochim. Biophys. Acta.*, **880**, 62.

DUNCAN, R., HUME, I.C., KOPECKOVA, P., ULBRICH, K., STROHALM, J. & KOPECEK, J. (1989). Anticancer agents coupled to N-(2-hydroxypropyl)methacrylamide copolymers, 3. Evaluation of adriamycin conjugates against mouse leukaemia L1210 *in vivo*. *J. Control. Release*, **10**, 51.

FALLON, R.J. & SCHWARTZ, A.L. (1989). Receptor-mediated delivery of drugs to hepatocytes. *Adv. Drug. Deliv. Rev.*, **4**, 49.

KERR, D.J. (1989). Intrahepatic arterial chemotherapy with cytotoxic drug-containing microspheres. *Br. J. Cancer*, **60**, 441.

KOOISTRA, T. (1979). *Endocytosis of proteins by liver cells*. PhD Thesis, Rijksuniversiteit Te Groningen.

NERENSTONE, S.R., IHDE, D.C. & FRIEDMAN, M.A. (1988). Clinical trials in primary hepatocellular carcinoma: current status and future directions. *Cancer Treat. Rev.*, **15**, 1.

O'HARE, K.B., HUME, I.C., SCARLETT, L. & 4 others (1989). Effect of galactose on interaction of N-(2-hydroxypropyl) methacrylamide copolymers with hepatoma cells in culture: preliminary applications to an anticancer agent, daunomycin. *Hepatology*, **10**, 207.

ORDER, S.E., KLEIN, J.L., ETTINGER, D., ALDERSON, P., SIEGELMAN, S. & LEICHNER, P. (1980). Phase I-II study of radiolabelled antibody integrated in the treatment of primary hepatic malignancies. *Int. J. Radiact. Oncol.*, **6**, 703.

PARDRIDGE, W.M., VAN HERLE, A.J., NARUSE, R.T., FIERER, G. & COSTIN, A. (1983). *In vivo* quantification of receptor-mediated uptake of asialoglycoproteins by rat liver. *J. Bio. Chem.*, **258**, 990.

PETERSON, G.L. (1983). Determination of total protein. *Meths. Enzymol.*, **91**, 95.

REJMANOVA, P., KOPECEK, J., DUNCAN, R. & LLOYD, J.B. (1985). Stability in plasma and serum of lysosomally degradable oligopeptide sequences in N-(2-hydroxypropyl) methacrylamide copolymers. *Biomaterials*, **6**, 45.

RIHOVA, B., ULBRICH, K., STROHALM, J. & 4 others (1989). Biocompatibility of N-(2-hydroxypropyl)methacrylamide copolymers containing adriamycin. *Biomaterials*, **10**, 335.

SCHLESINGER, P.H., RODMAN, J.S., DOEBBER, T.W. & 4 others (1980). The role of extra-hepatic tissues in the receptor-mediated plasma clearance of glycoprotein terminated by mannose or N-acetylglucosamine. *Biochem. J.*, **192**, 597.

SCHNEIDER, Y.-J., ABARCA, J., ABOUD, PIRAK, E. & 8 others (1984). Drug targeting in human cancer chemotherapy. In *Receptor-Mediated Targeting of Drugs*, p.1, Gregoriadis, G., Poste, G., Senior, J. & Trouet, A. (eds). NATO ASI Series A: Life Sciences Volume 82, Plenum Press: New York.

SCHWARTZ, A.L., FRIDOVICH, S.E. & LODISH, H.K. (1982). Kinetics of internalization and recycling of the asialoglycoprotein receptor in a hepatoma cell line. *J. Biol. Chem.*, **257**, 4230.

SEYMOUR, L.W., DUNCAN, R., STROHALM, J. & KOPECEK, J. (1987). Effect of molecular weight (Mw) of N-(2-hydroxypropyl) methacrylamide copolymers on body distribution and rate of excretion after subcutaneous, intraperitoneal and intravenous administration to rats. *J. Biomed. Mater. Res.*, **21**, 1341.

SEYMOUR, L.W., ULBRICH, K., STROHALM, J. & DUNCAN, R. (1990). Pharmacokinetics of polymer-bound adriamycin. *Biochem. Pharm.*, **39**, 1125.

SHAPIRA, J., GOTFRIED, M., LISHNER, M. & RAVID, M. (1990). Reduced cardiotoxicity of doxorubicin by a 6-hour infusion regimen. *Cancer*, **65**, 870.

STROHALM, J. & KOPECEK, J. (1978). Poly N-(2-hydroxypropyl) methacrylamide IV. Heterogenous polymerization. *Angew. Makromol. Chem.*, **70**, 109.

TROUET, A. (1974). Isolation of modified liver lysosomes. *Meths. Enzymol.*, **31**, 323.

VERA, D.R., STADALNICK, R.C. & KROHN, K.A. (1985). Technetium-99m galactosyl-neoglycoalbumin: preparation and preclinical studies. *J. Nucl. Med.*, **26**, 57.

VIRGOLINI, I., MULLER, C., KLEPETKO, W. & 4 others (1990). Decreased hepatic function in patients with hepatoma or liver metastasis monitored by a hepatocyte specific galactosylated radioligand. *Br. J. Cancer*, **61**, 937.

WARREN, D.R. & DOYLE, D. (1981). Turnover of the surface proteins and the receptor for serum asialoglycoproteins in primary cultures of rat hepatocytes. *J. Biol. Chem.*, **256**, 1346.

WILLIAMS, K.E., KIDSTON, E.M., BECK, F. & LLOYD, J.B. (1975*a*). Quantitative studies of pinocytosis. 1. Kinetics of uptake of [$^{125}$I] polyvinylpyrrolidone by rat yolk sac cultured *in vitro*. *J Cell. Biol.*, **64**, 113.

WILLIAMS, K.E., KIDSTON, E.M., BECK, F. & LLOYD, J.B. (1975*b*). Quantitative studies of pinocytosis. II. Kinetics of protein uptake and digestion by rat yolk sac cultured *in vivo*. *J. Cell. Biol.*, **64**, 123.

YEUNG, T.K., SIMMONDS, R.H., HOPEWELL, J.W., SEYMOUR, L.W., DUNCAN, R. & ULBRICH, K. (1989). Comparative toxicity of HPMA copolymer-adriamycin conjugates and free adriamycin. *Br. J. Cancer*, **60**, 446.

ZILVA, J.F. & PANNALL, P.R. (1981). *Clinical Chemistry in Diagnosis and Treatment*. 3rd Ed. Lloyd-Lake Medical Books Ltd, London, UK.